# Real-Time AI-Based Assessment of Glottic Lesions During Flexible Laryngoscopy: Comparison with Expert Evaluation and Histopathology

**DOI:** 10.3390/medsci14040458

**Published:** 2026-08-06

**Authors:** Johannes Kränzlein, Konstantinos Anagnostopoulos, Christoph Arens, Nikolaos Davaris

**Affiliations:** Department of Otorhinolaryngology, Head and Neck Surgery and Plastic Surgery, University Hospital Giessen & Marburg, Giessen Campus, 35392 Giessen, Germany; konstantinos.anagnostopoulos@hno.med.uni-giessen.de (K.A.); christoph.arens@hno.med.uni-giessen.de (C.A.); nikolaos.davaris@hno.med.uni-giessen.de (N.D.)

**Keywords:** laryngeal carcinoma, flexible laryngoscopy, artificial intelligence, computer-aided diagnosis, narrow-band imaging, optical biopsy, real-time endoscopy, diagnostic accuracy, head and neck cancer

## Abstract

**Background:** Endoscopic risk assessment of glottic lesions is examiner-dependent. This prospective single-center diagnostic evaluation study assessed the real-time analytical performance of the CE-MDR Class IIb-certified Zeno AI system during structured flexible laryngoscopy. **Methods:** A total of 109 patients underwent protocol-based flexible laryngoscopy. Post-epiglottic visualization of the glottic level and additional acquisition characteristics were documented systematically. Three experienced examiners independently classified each case as morphologically unremarkable, benign, or malignant/suspicious; majority vote served as the expert reference. Zeno AI classified findings as unremarkable, benign, malignant, or uncertain. The system was evaluated in shadow mode, and AI outputs were included only when the same classification remained stable for at least three seconds. The primary analysis assessed agreement between Zeno AI and the expert majority using Cohen’s κ. Interobserver agreement among the examiners was assessed using Fleiss’ κ. Diagnostic performance against histopathology was evaluated secondarily in operated patients using a primary intent-to-diagnose analysis and a secondary definitive-output analysis. **Results:** Expert majority classified 64/109 findings as unremarkable, 34/109 as benign, and 11/109 as malignant/suspicious. AI classified 65/109 as unremarkable, 26/109 as benign, 12/109 as malignant, and 6/109 as uncertain. Excluding uncertain outputs, AI and the expert majority agreed in 99/103 cases (96.1%; κ = 0.927). Histopathology was available in 36 patients, with 27 findings negative for malignancy and 9 positive for malignancy. In the primary intent-to-diagnose analysis, treating uncertain outputs as test-negative, sensitivity was 88.9%, specificity 92.6%, PPV 80.0%, and NPV 96.2%. In the secondary analysis restricted to definitive AI outputs, sensitivity was 100%, specificity 91.7%, PPV 80.0%, and NPV 100%. No finding positive for malignancy was classified as benign; one high-grade dysplasia was classified as uncertain. **Conclusions:** Zeno AI showed high agreement with expert assessment during structured flexible laryngoscopy. However, its potential clinical benefit was not evaluated in this shadow-mode study. Uncertain outputs should be regarded as non-definitive and require expert assessment, while histopathology remains essential for definitive diagnosis.

## 1. Introduction

Laryngeal carcinomas are among the most common malignancies of the head and neck region. In Germany, approximately 3200 new cases are registered each year, and worldwide estimates indicate approximately 190,000 new cases of laryngeal cancer in 2024 [1,2]. Prognosis is strongly stage dependent. Early-stage glottic carcinomas are associated with favorable oncological outcomes and can often be treated with organ-preserving approaches, whereas advanced-stage disease is associated with a substantially poorer prognosis [3]. Therefore, early detection and reliable risk assessment of glottic lesions remain central objectives in laryngological oncology.

Flexible laryngoscopy is an essential diagnostic procedure in routine otorhinolaryngological practice. It enables real-time visualization of the laryngeal structures and allows assessment of vocal fold morphology, mobility, mucosal surface changes, and suspicious lesions. Modern flexible endoscopy has evolved from fiberoptic visualization to high-resolution digital video endoscopy and represents an indispensable diagnostic tool in contemporary otorhinolaryngology [4]. Flexible laryngoscopy can be performed under white light and complemented by narrow-band imaging (NBI), which enhances visualization of subepithelial vascular patterns [5]. Previous work has shown that flexible transnasal endoscopy using white-light imaging and NBI provides relevant diagnostic information for the assessment of laryngeal malignancy, but that interpretation is subject to interobserver variability and may be influenced by previous laryngeal surgery [5].

At the glottic level, NBI can support the differentiation of benign, premalignant, and malignant lesions by improving the assessment of superficial vascular patterns. The European Laryngological Society (ELS) proposed a descriptive classification of superficial vascular changes in the vocal folds, distinguishing longitudinal from perpendicular vascular changes. Longitudinal vascular changes include ectasia, increased vessel density, increased branching, meandering, and changes in vessel direction. These changes remain predominantly within the plane of the vocal fold and are generally considered non-suspicious, although they may occur in association with mechanical stress, inflammation, or benign vocal fold lesions. In contrast, perpendicular vascular changes extend toward the epithelial surface and may appear as intraepithelial papillary capillary loops. Such patterns can reflect neoangiogenesis in dysplastic and malignant lesions. However, perpendicular vascular changes may also occur in benign exophytic lesions, particularly papillomas, in which the vascular loops typically show wide-angled turning points [6]. This classification was subsequently validated in a retrospective multicenter study including 707 patients, which reported high interobserver agreement and relevant diagnostic performance for the detection of squamous intraepithelial neoplasia and squamous cell carcinoma. Interobserver agreement was high, with κ values of 0.954 at one center and 0.872 at the other. For the detection of squamous cell carcinoma, sensitivity was 90%, specificity 58%, positive predictive value 56%, and negative predictive value 91% [7].

The diagnostic value of flexible laryngoscopy depends not only on the imaging modality itself, but also on the quality and standardization of image acquisition. Modern flexible video endoscopy enables high-resolution visualization and documentation of laryngeal structures, while technical parameters such as resolution, field of view, angulation, and depth of field influence whether subtle mucosal changes, pathological vascular patterns, and minute early-stage carcinomas can be reliably detected [4]. In flexible transnasal laryngoscopy, image magnification can be improved by decreasing the distance between the endoscope tip and the vocal folds, and specific maneuvers such as dipping and rotation have been described to improve visualization of the vocal folds, anterior commissure, inferior vocal fold surface, Morgagni’s ventricle, and other difficult-to-assess glottic regions [8]. Observer studies further indicate that visualization technology and examiner experience influence reliable assessment of pharyngeal and laryngeal lesions, with high-definition laryngoscopy and NBI improving diagnostic reliability [9]. These acquisition-related factors are particularly relevant for AI-based real-time video analysis. Recent work on automated laryngoscopic image-quality assessment has shown that large endoscopic datasets may contain a high proportion of low-quality or irrelevant frames, which can hinder clinical and research use [10]. Therefore, consistent visualization of the vocal folds and standardized acquisition of diagnostically evaluable frames are important prerequisites for robust AI-based lesion detection and classification.

Artificial intelligence has become increasingly relevant in medical image analysis and endoscopy. In gastrointestinal endoscopy, computer-assisted detection and classification systems have shown promising results in the detection and characterization of colorectal lesions, and prospective studies have demonstrated that AI-based systems can increase polyp and adenoma detection rates during colonoscopy [11,12,13]. In laryngology, however, AI-based systems are not yet established in routine clinical practice. A systematic review and meta-analysis summarized early evidence on AI applications in laryngeal endoscopy and highlighted the potential of AI-based approaches for lesion detection and classification [14]. Initial studies have shown that deep learning models can classify laryngeal lesions based on endoscopic images or video sequences. Wellenstein et al. demonstrated an AI-based system for the detection of laryngeal carcinoma during endoscopy, showing the potential for real-time detection [15]. He et al. developed a deep convolutional neural network for the diagnosis of laryngeal squamous cell carcinoma and reported high diagnostic performance [16]. Further recent work has addressed segmentation and dataset development for laryngeal lesion assessment, reflecting the increasing relevance of structured endoscopic image data for AI-based applications [17,18].

Building on the detection, classification, and segmentation approaches investigated in previous studies, future AI-assisted laryngoscopy systems may contribute to a more standardized assessment of glottic findings across different levels of examiner experience. Potential applications include real-time second-reader support, risk stratification, prioritization for specialist evaluation, and identification of suspicious areas for targeted biopsy. However, these potential clinical applications have not yet been established in routine practice and require prospective interventional validation [9,14,15,16,17,18].

The Zeno AI system (WSK Medical, Amsterdam, The Netherlands) is a CE-MDR Class IIb-certified AI system designed for real-time detection, localization, and classification of glottic lesions during laryngoscopic video examination. The system analyzes endoscopic video material in real time and classifies detected abnormalities as benign, malignant, or uncertain. It was developed to support the endoscopic assessment of glottic pathologies, particularly in settings where examiner experience may vary. However, prospective clinical data on its performance under routine flexible laryngoscopy conditions remain limited. It remains unclear how the system performs when applied within a structured flexible laryngoscopy protocol for glottic lesion assessment and compared with experienced laryngological examiners and histopathology.

The primary aim of this prospective single-center diagnostic evaluation study was to assess agreement between the Zeno AI system and experienced examiners during structured flexible laryngoscopy for glottic lesion assessment. A secondary aim was to evaluate the diagnostic performance of AI-based and examiner-based classification against histopathology in the subgroup of patients who underwent microlaryngoscopy with tissue sampling or resection.

## 2. Materials and Methods

### 2.1. Study Design and Setting

This prospective single-center diagnostic evaluation pilot study was conducted at the Department of Otorhinolaryngology, Head and Neck Surgery and Plastic Surgery of a tertiary university hospital in Germany. Consecutive enrolled patients consented to real-time AI analysis during flexible laryngoscopy were screened for inclusion between February 2026 and June 2026.

All study participants were informed about the objective and procedure of the planned examination and provided written informed consent before inclusion. The study was conducted in accordance with the Declaration of Helsinki and was approved by the local Ethics Committee [Approval number 79/26; date of approval: 2 July 2026].

The study evaluated the Zeno AI system within a structured flexible laryngoscopy protocol for glottic lesion assessment. The statistical analysis was restricted exclusively to the glottic level. Pharyngeal, supraglottic, and subglottic abnormalities were not included in the statistical evaluation.

### 2.2. Patient Recruitment and Eligibility Criteria

**Figure 1:**
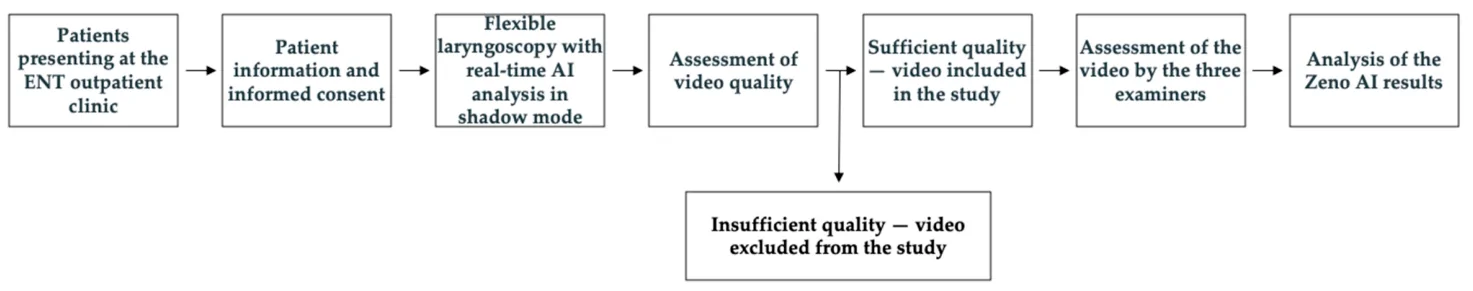
Study workflow.

Consecutive consenting patients were eligible for inclusion if a flexible laryngoscopic video examination of the glottic level was available for expert-based and AI-based assessment. Flexible laryngoscopy was performed as part of routine clinical assessment according to the individual clinical indication. Study inclusion depended on informed consent and the availability of the Zeno AI system; patients were not preselected based on symptoms or suspicion of a glottic lesion. Video recordings were excluded if the glottic level could not be identified or if image quality was insufficient for reliable assessment because of severe motion blur, defocusing, overexposure, mucus, incomplete visualization of the vocal folds, or other artifacts. The study workflow is shown in Figure 1. In total, 109 patients fulfilled the inclusion criteria and were included in the final analysis. Demographic and clinical cohort characteristics, including age, sex, and previous glottic surgery, were recorded from patient history and available medical records.

### 2.3. Structured Flexible Laryngoscopy Protocol for Glottic Lesion Assessment

Flexible laryngoscopy was performed using the VISERA ELITE III CLL-S700 light source, the VISERA ELITE III OTV-S700 video processor, and the ENF-VH flexible endoscope (Olympus, Tokyo, Japan). The examination protocol aimed to obtain standardized high-quality visualization of the glottic level suitable for both examiner-based and AI-based assessment. The minimum acquisition requirement for inclusion was an evaluable view of the glottic level that allowed reliable assessment of both vocal folds. White-light imaging was performed in all included cases. Narrow-band imaging was obtained where technically feasible and was considered part of the assessment when an evaluable NBI sequence of the glottic lesion or glottic level was available for examiner-based classification. In patients with glottic lesions, additional structured acquisition maneuvers were attempted where anatomically and technically feasible. A near-contact view was defined as a flexible laryngoscopic view in which the endoscope tip was advanced as close as technically feasible to the glottic lesion without intentional mucosal contact. A rotated/inverted glottic view was defined as an additional maneuver in which the flexible endoscope was rotated to provide an altered perspective of the glottic lesion, particularly of regions that may be difficult to assess from the standard view, such as the anterior commissure or the inferior vocal fold surface. In cases where microlaryngoscopy was indicated, flexible laryngoscopy was performed within two weeks prior to surgery. Flexible laryngoscopy was performed by several examiners, each of whom had completed at least two years of clinical training in otorhinolaryngology. The use of multiple examiners was intended to reflect routine clinical practice and to reduce dependence on a single examination technique. The duration of the recorded video sequences varied according to the clinical indication, patient tolerance, anatomical conditions, and the presence or absence of a glottic lesion.

For each included video, acquisition characteristics were documented, including the availability of post-epiglottic visualization, evaluable white-light imaging, evaluable NBI sequences, near-contact imaging, and rotated/inverted glottic visualization. Acquisition characteristics were summarized for the full cohort and reported separately for lesion-positive cases and for the histopathology subgroup.

### 2.4. AI System and Real-Time Video Analysis

The CE-MDR Class IIb-certified Zeno AI system, version 1.4.0 (WSK Medical), was used for real-time analysis of the laryngoscopic video stream. The system is a deep learning-based object detection and classification model designed to identify, localize, and classify glottic lesions in laryngoscopic videos. The model used in this study represents an updated version of the system described by Wellenstein et al. and was trained on endoscopic images [15].

The AI pipeline consists of two sequential components. First, glottic lesions are detected using a YOLOv8-based object detection algorithm. Predicted bounding boxes with a confidence threshold below 0.2 were discarded. Second, detected lesions were classified using an EfficientNet-B0 convolutional neural network as benign or malignant. To account for prediction uncertainty, a predefined classification confidence threshold was applied. AI outputs meeting or exceeding this threshold were assigned a definitive classification, while outputs below this threshold were classified as uncertain. This threshold was determined before the start of the study using a held-out validation dataset and was not adjusted during the study.

AI outputs were logged automatically throughout each examination. The examining physician was not shown the AI output during the procedure. Therefore, this study evaluated the real-time analytical performance of the AI system in a shadow-mode setting and did not assess the direct influence of AI output on clinical decision-making. The AI analysis was performed independently of, and prior to, histopathological assessment.

For statistical comparison, an AI output was included only if the system provided a continuous and identical classification for at least three seconds. This criterion was applied to reduce the influence of transient artifacts caused by camera movement, mucus, brief loss of focus, or overexposure. For the three-category agreement analysis between Zeno AI and the expert majority assessment, cases classified as uncertain were excluded because the expert evaluators did not use an equivalent uncertain category. These cases were reported separately as non-definitive AI outputs. For diagnostic performance against histopathology, uncertain outputs were included in the primary intent-to-diagnose analysis and treated as test-negative for malignancy detection.

Further information on the development and internal validation of the AI system, including a reference to a publication by the developers, is provided in the Supplementary Materials (S4).

### 2.5. Expert Assessment

The laryngoscopic videos were independently evaluated by three experienced examiners. The examiners assessed the glottic level and documented whether a glottic lesion was present. For classification, three categories were available: morphologically unremarkable vocal folds, benign glottic lesion, or malignant/suspicious glottic lesion. A lesion was classified as malignant/suspicious when the examiner considered squamous cell carcinoma, carcinoma in situ, or high-grade dysplasia to be a relevant endoscopic diagnosis.

The examiners evaluated the videos independently and were blinded to both the AI output and the histopathological results. Classification was based on individual clinical judgment, taking into account overall lesion morphology, surface characteristics, and vascular patterns, with reference to the ELS classification of superficial vascular changes in the vocal folds. No formal standardized scoring system was applied. In cases of disagreement, the majority vote was used as the expert reference for agreement analysis. In addition, a specific suspected diagnosis was documented for descriptive analysis.

### 2.6. Histopathological Reference Standard

Patients with a clinical indication for surgery underwent microlaryngoscopy with tissue sampling or resection according to standard clinical practice. Histopathological findings from these procedures served as the reference standard for diagnostic performance analysis. Histopathology was available in 36 cases. The microlaryngoscopy was performed after the assessment of the three examiners and the AI.

Histopathological findings were reported according to the current WHO Classification of Head and Neck Tumours [19]. For statistical analysis, findings were dichotomized as negative or positive for malignancy. Benign lesions and low-grade dysplasia were categorized as negative for malignancy, whereas high-grade dysplasia, carcinoma in situ, and invasive squamous cell carcinoma were categorized as positive for malignancy. Low-grade dysplasia was retained as a precursor lesion in the descriptive reporting but assigned to the negative-for-malignancy group for the binary analysis because of its comparatively low malignant transformation risk.

### 2.7. Endpoints

The primary endpoint was agreement between Zeno AI and the expert majority assessment for glottic lesion detection and three-category classification in patients with definitive AI output. Lesion detection was defined as classification as benign or malignant/suspicious by expert majority assessment and as definitive benign or malignant output by Zeno AI.

Secondary endpoints included interobserver agreement among the three experienced examiners, the frequency of uncertain AI outputs, pairwise agreement between individual examiners and Zeno AI, and the diagnostic performance of AI-based and examiner-based classification against histopathology in the surgical subgroup. The primary diagnostic performance analysis against histopathology followed an intent-to-diagnose approach and included all patients with available histopathology. Uncertain AI outputs were treated as test-negative for malignancy detection. A secondary analysis was restricted to cases with definitive AI output.

Endoscopic acquisition characteristics were analyzed descriptively. These included the availability of post-epiglottic visualization, evaluable white-light imaging, evaluable NBI sequences, near-contact imaging, and rotated/inverted glottic visualization.

### 2.8. Analysis Sets and Reference Standards

Three analysis sets were defined. The full cohort included all patients with evaluable flexible laryngoscopic video data and was used for cohort description, acquisition characteristics, expert majority assessment, AI output distribution, and AI–expert majority agreement. The lesion-positive subset included all cases classified as showing a glottic lesion by expert majority assessment and was used for descriptive analysis of lesion-specific suspected diagnoses, acquisition characteristics, and discordant or non-definitive AI outputs. The lesion-positive subset was derived from the full cohort and was larger than the histopathology subgroup because not all patients with benign-appearing lesions underwent surgery. The histopathology subgroup included all patients with available histopathological reference standard and was used for diagnostic performance analyses against histopathology.

In the full cohort and lesion-positive subset, analyses were restricted to agreement between AI output and expert-based assessment because histopathology was not available for all patients. For these analyses, the majority assessment of the three experienced examiners served as the clinical reference for lesion detection and three-category classification. Diagnostic performance metrics were calculated only in the histopathology subgroup, in which histopathological findings served as the reference standard.

### 2.9. Statistical Analysis

Statistical analysis was performed using Python 3.12 with pandas for data management, NumPy and SciPy for descriptive and inferential statistical calculations, and scikit-learn for agreement analyses. Cohort characteristics were summarized descriptively. Continuous variables were tested for normal distribution using the Shapiro–Wilk test with a significance level of α = 0.05.

Interobserver agreement among the three examiners was assessed using Fleiss’ κ. Agreement between AI-based classification and the expert majority assessment was assessed using Cohen’s κ. In the primary agreement analysis, cases classified as uncertain by the AI system were excluded. The frequency and proportion of uncertain AI outputs were reported separately.

Pairwise Cohen’s κ values were additionally calculated between the individual examiners and between each examiner and Zeno AI in the histopathology subgroup with definitive AI output. Cases classified as uncertain by Zeno AI were excluded from this pairwise analysis to ensure comparison of the same three-category classification task across raters. These pairwise κ values were visualized as a heatmap.

The interpretation of κ values followed Landis and Koch: <0.00, poor; 0.00–0.20, slight; 0.21–0.40, fair; 0.41–0.60, moderate; 0.61–0.80, substantial; and 0.81–1.00, almost perfect agreement [20].

In the subgroup of patients with available histopathology, diagnostic performance was calculated for the AI system and for examiner-based assessment using histopathological classification as the reference standard. Sensitivity, specificity, positive predictive value, and negative predictive value were calculated with corresponding 95% confidence intervals. Confidence intervals for proportions were calculated using the Wilson score method. Confidence intervals for Cohen’s κ were calculated using the asymptotic standard error method according to Fleiss, Cohen, and Everitt.

For AI-based diagnostic performance, the primary intent-to-diagnose analysis included all patients with available histopathology. Uncertain AI outputs were treated as test-negative for malignancy detection. A secondary definitive-output analysis was restricted to cases in which Zeno AI provided a definitive classification.

In an exploratory analysis restricted to lesion-positive cases, a lesion-specific acquisition score was calculated by assigning one point each for the availability of an evaluable NBI sequence, near-contact imaging, and rotated/inverted glottic visualization. The score ranged from 0 to 3. Acquisition scores were compared between cases with definitive Zeno AI output and cases with uncertain Zeno AI output using the Mann–Whitney U test. In addition, cases with complete lesion-specific acquisition, defined as availability of all three features, were compared with cases with incomplete acquisition using Fisher’s exact test. These analyses were considered exploratory because of the limited number of uncertain outputs.

## 3. Results

### 3.1. Patient and Cohort Characteristics

The final cohort comprised 109 patients, including 63/109 male patients (57.8%) and 46/109 female patients (42.2%). The mean age was 61.3 years, with a range of 4 to 86 years. The lesion-positive subgroup comprised 45 patients, including 30 male patients (66.7%) and 15 female patients (33.3%). The mean age in this subgroup was 59.1 years, with a range of 4 to 84 years. The histopathology subgroup comprised 36 patients, including 25/36 male patients (69.4%) and 11/36 female patients (30.6%). The mean age in this subgroup was 59.7 years, with a range of 4 to 84 years. Previous glottic surgery was documented in 23/45 lesion-positive cases (51.1%) and in 19/36 cases (52.8%) of the histopathology subgroup. Patient and cohort characteristics are summarized in Table 1.

By expert majority assessment, 64/109 cases (58.7%) were classified as morphologically unremarkable, whereas 45/109 cases (41.3%) showed a glottic lesion.

### 3.2. Endoscopic Acquisition Characteristics

In the full cohort, post-epiglottic visualization of the glottic level was available in 106/109 videos (97.2%). Near-contact imaging was available in 60/109 cases (55.0%), rotated/inverted glottic visualization in 52/109 cases (47.7%), and evaluable NBI sequences in 65/109 cases (59.6%).

Among the 45 lesion-positive cases, post-epiglottic close-up visualization was available in all cases. Near-contact imaging was available in 38/45 cases (84.4%), rotated/inverted glottic visualization in 31/45 cases (68.9%), and evaluable NBI sequences in 41/45 cases (91.1%).

In the histopathology subgroup, post-epiglottic visualization was available in all 36 cases (100%). Near-contact imaging was available in 31/36 cases (86.1%), rotated/inverted glottic visualization in 26/36 cases (72.2%), and evaluable NBI sequences in 35/36 cases (97.2%). Endoscopic acquisition characteristics are summarized in Table 2.

### 3.3. Expert-Based Assessment and Interobserver Agreement

By expert majority assessment, 64/109 findings (58.7%) were classified as morphologically unremarkable, 34/109 findings (31.2%) as benign, and 11/109 findings (10.1%) as malignant/suspicious. Accordingly, the expert majority detected a glottic lesion in 45/109 cases (41.3%).

All 64/109 morphologically unremarkable cases were classified identically by all three examiners. Among the 45 lesion-positive cases, complete agreement among all three examiners was observed in 35/45 cases (77.8%), whereas a majority decision was required in 10/45 cases (22.2%). In the full cohort, complete agreement was observed in 99/109 cases (90.8%).

Interobserver agreement among the three examiners was almost perfect in the full cohort for three-category classification (Fleiss’ κ = 0.885; 95% CI, 0.814–0.947) and for lesion detection (Fleiss’ κ = 0.910). In the lesion-positive subset, interobserver agreement for three-category classification was substantial (Fleiss’ κ = 0.660). In the histopathology subgroup, complete agreement was observed in 30/36 cases (83.3%), with substantial interobserver agreement for three-category classification (Fleiss’ κ = 0.743).

**Figure 2:**
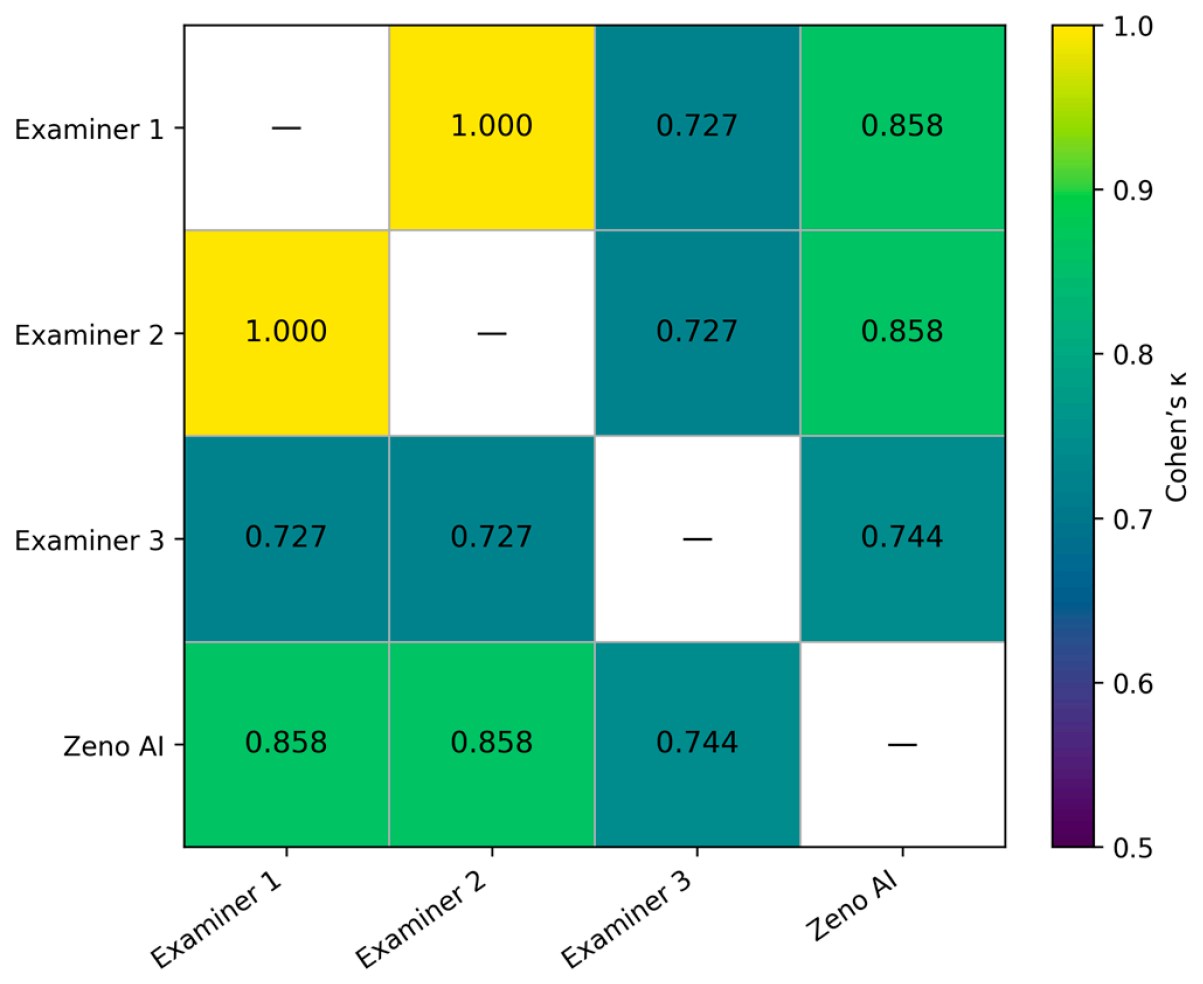
Pairwise agreement between the three experienced examiners and Zeno AI in the histopathology subgroup. The heatmap shows pairwise Cohen’s κ values for three-category classification in cases with definitive AI output (n = 32). Cases with uncertain AI output were excluded.

To further illustrate agreement in the clinically most relevant subgroup, pairwise Cohen’s κ values between the individual examiners and Zeno AI in the histopathology subgroup with definitive AI output are visualized in Figure 2.

Among the 34/45 benign lesions by expert majority assessment, the most frequent suspected diagnoses were papilloma (n = 12/34; 35.3%), hyperkeratosis (n = 6/34; 17.6%), vocal fold cyst (n = 6/34; 17.6%), vocal fold polyp (n = 3/34; 8.8%), and Reinke’s edema (n = 3/34; 8.8%). Less frequent suspected benign diagnoses included granuloma, hyperplasia, granulation tissue, and suspected laryngeal amyloidosis. Among the 11/45 malignant/suspicious cases, the suspected diagnoses were squamous cell carcinoma (n = 9/11; 81.8%), carcinoma in situ (n = 1/11; 9.1%), and high-grade dysplasia (n = 1/11; 9.1%).

### 3.4. AI-Based Assessment and Agreement with Expert Majority

The Zeno AI system classified 65/109 findings (59.6%) as morphologically unremarkable, 26/109 findings (23.9%) as benign, and 12/109 findings (11.0%) as malignant. Thus, Zeno AI provided a definitive lesion-positive output in 38/109 cases (34.9%). In 6/109 cases (5.5%), the AI output was classified as uncertain. Among cases with definitive AI output, the AI lesion detection rate was 38/103 (36.9%). The distribution of expert majority and AI classifications is shown in Table 3.

Cases classified as uncertain were excluded from the primary AI–expert agreement analysis. In the remaining 103 cases with definitive AI output, Zeno AI and expert majority assessment agreed in 99/103 cases (96.1%; 95% CI, 90.4–98.5), corresponding to almost perfect agreement (Cohen’s κ = 0.927; 95% CI, 0.857–0.996). For lesion detection, Zeno AI and expert majority assessment agreed in 101/103 cases (98.1%; Cohen’s κ = 0.959). The cross-tabulation of expert majority and definitive Zeno AI classifications is presented in Table 4. Zeno AI did not provide lesion-specific diagnostic categories beyond benign, malignant and uncertain in the present cohort. Representative examples of definitive benign Zeno AI outputs are provided in Supplementary Videos S1 and S2, showing a glottic papilloma (S1) and vocal fold polyps (S2), respectively.

### 3.5. Discordant and Non-Definitive AI Outputs

In total, 10/109 cases showed either discordant definitive AI output or non-definitive AI output. Among the 45 lesion-positive cases, four definitive AI outputs were discordant with the expert majority assessment. Two benign lesions were classified as malignant by Zeno AI; both cases were clinically classified as hyperkeratosis by expert assessment. Two benign lesions were not detected by Zeno AI and were classified as morphologically unremarkable; these included one vocal fold cyst and one papilloma.

Five lesion-positive cases were classified as uncertain by Zeno AI. Four of these were classified as benign by expert majority assessment, including two papillomas, one vocal fold polyp, and one lesion with hyperplastic vessels. One lesion classified as malignant/suspicious by expert majority assessment was classified as uncertain by Zeno AI. In addition, one morphologically unremarkable case in the full cohort was classified as uncertain by Zeno AI. Discordant and non-definitive AI outputs are listed in Table 5.

In an exploratory analysis restricted to lesion-positive cases, the lesion-specific acquisition score did not differ significantly between cases with definitive Zeno AI output and cases with uncertain output. The median acquisition score was 3 (IQR, 2–3) in cases with definitive output and 3 (IQR, 1–3) in cases with uncertain output (*p* = 0.646). Complete lesion-specific acquisition was not associated with a lower frequency of uncertain output (3/28, 10.7% vs. 2/17, 11.8%; Fisher’s exact test, *p* = 1.000). Given the small number of uncertain outputs, this analysis was underpowered and should be interpreted with caution.

### 3.6. Histopathology Subgroup

Microlaryngoscopy with tissue sampling or resection was indicated in 37/109 patients (33.9%). No patient with morphologically unremarkable vocal folds underwent surgery. Among the 37 patients with surgical indication, one patient died of an unrelated illness before the planned procedure; therefore, histopathology was available in 36/109 cases.

Of the 34 benign cases by expert majority assessment, 26 underwent surgery (76.5%). All 11 cases classified as malignant/suspicious by expert majority assessment had an indication for surgery; one of these patients died before the planned procedure. Histopathological classification of the 36 operated cases revealed 27 findings negative for malignancy (75.0%) and 9 findings positive for malignancy (25.0%). Histopathological findings are shown in Table 6.

### 3.7. Diagnostic Performance Compared with Histopathology

Compared with histopathology, the expert majority assessment classified all 9/36 findings positive for malignancy as malignant/suspicious. One finding negative for malignancy, corresponding to low-grade dysplasia, was classified as malignant/suspicious by expert majority assessment. This resulted in a sensitivity of 100% (95% CI, 70.1–100), specificity of 96.3% (95% CI, 81.7–99.3), PPV of 90.0% (95% CI, 59.6–98.2), NPV of 100% (95% CI, 87.1–100), and Cohen’s κ = 0.929 (95% CI, 0.791–1.000).

For the primary intent-to-diagnose analysis of Zeno AI performance, all 36 patients with available histopathology were included. Uncertain Zeno AI outputs were treated as test-negative for malignancy detection. Under this assumption, one histologically confirmed high-grade dysplasia classified as uncertain by Zeno AI was counted as false-negative. Sensitivity was 88.9% (95% CI, 56.5–98.0), specificity was 92.6% (95% CI, 76.6–97.9), PPV was 80.0% (95% CI, 49.0–94.3), NPV was 96.2% (95% CI, 81.1–99.3), and Cohen’s κ was 0.786 (95% CI, 0.555–1.000). The high-grade dysplasia classified as uncertain by Zeno AI is shown in Supplementary Video S3.

In a secondary analysis restricted to the 32 cases with definitive AI output, Zeno AI correctly classified all 8 histologically positive findings as malignant. Two findings negative for malignancy were classified as malignant by Zeno AI. This resulted in a sensitivity of 100% (95% CI, 67.6–100), specificity of 91.7% (95% CI, 74.2–97.7), PPV of 80.0% (95% CI, 49.0–94.3), NPV of 100% (95% CI, 85.1–100), and Cohen’s κ = 0.846 (95% CI, 0.642–1.000).

Diagnostic performance metrics are summarized in Table 7. Given the limited size of the histopathology subgroup and the small number of findings positive for malignancy, these estimates were associated with wide confidence intervals and should be interpreted with caution. The classification of one high-grade dysplasia as uncertain demonstrates that uncertain outputs should be regarded as non-definitive and require further expert assessment.

## 4. Discussion

### 4.1. Principal Findings

This prospective single-center diagnostic evaluation pilot study assessed the real-time analytical performance of the Zeno AI system within a structured flexible laryngoscopy protocol for glottic lesion assessment. In the full cohort, Zeno AI showed high agreement with the expert majority assessment. In the histopathology subgroup, diagnostic performance estimates of Zeno AI were in the range of the expert majority assessment when only definitive AI outputs were considered. Importantly, no histopathologically confirmed malignant lesion was classified as benign by Zeno AI. However, one histologically confirmed high-grade dysplasia was classified as uncertain, demonstrating that non-definitive AI outputs should not be interpreted as reassuring and must prompt further expert evaluation.

These findings support the feasibility of real-time AI analysis during flexible laryngoscopy, but they also underline important current limitations. The evaluated version of the system provided benign–malignant classification and an uncertain category but did not provide sufficiently differentiated lesion-specific diagnostic categories in the present cohort. Therefore, its potential clinical role should be understood as supportive risk stratification rather than autonomous diagnostic decision-making or optical biopsy.

### 4.2. Clinical Context: Flexible Laryngoscopy and Examiner-Dependent Assessment

The present findings should be interpreted in the context of established flexible laryngoscopy without AI support. Flexible transnasal laryngoscopy is widely used for the evaluation of laryngeal lesions, and NBI can improve visualization of superficial vascular patterns relevant to the differentiation of benign, premalignant, and malignant lesions. However, examiner-based interpretation remains influenced by observer variability, image quality, lesion morphology, previous laryngeal surgery, and clinical experience [5]. This is particularly relevant because, in the full cohort of the present study, the expert majority assessment served as the clinical reference standard for AI agreement analyses.

The diagnostic relevance of NBI is well established independently of AI. The ELS classification provides a standardized framework for interpreting superficial vascular changes in the vocal folds, and multicenter validation data have demonstrated relevant diagnostic performance for detecting squamous intraepithelial neoplasia and squamous cell carcinoma [6,7]. Meta-analytic and systematic review data further support the diagnostic value of NBI in laryngeal cancer and preneoplastic laryngeal lesions [21,22]. Against this background, AI-based interpretation should be viewed as an additional computational layer on top of an already complex and examiner-dependent endoscopic diagnostic process.

### 4.3. Structured Flexible Laryngoscopy and Acquisition Quality

A central methodological aspect of the present study is the use of a structured flexible laryngoscopy protocol. The performance of AI-based video analysis depends not only on the algorithm but also on the diagnostic quality of the visual input. In flexible transnasal laryngoscopy, close-up visualization and maneuvers such as dipping and rotation may improve visualization of difficult-to-assess regions, including the anterior commissure and the inferior vocal fold surface [8]. These considerations provided the rationale for documenting post-epiglottic visualization, NBI availability, near-contact imaging, and rotated/inverted views.

However, the documented acquisition characteristics were analyzed descriptively and exploratorily only. In lesion-positive cases, a lesion-specific acquisition score was not significantly associated with definitive versus uncertain Zeno AI output. This finding should be interpreted cautiously because uncertain outputs were infrequent and the study was not powered to determine the effect of individual acquisition maneuvers on AI performance. Therefore, the present data should not be interpreted as evidence for or against the diagnostic superiority of any specific acquisition technique.

Nevertheless, acquisition quality remains relevant for real-time AI systems because motion blur, mucus, overexposure, incomplete visualization, or rapid camera movement may cause fluctuating or non-definitive outputs [10,23,24]. For this reason, recordings with insufficient image quality were excluded, and Zeno AI classifications were included in the primary analysis only when the same classification remained stable for at least three seconds.

### 4.4. Comparison with Previous AI Studies in Laryngology

Previous studies have shown that AI-based approaches can support the assessment of laryngeal lesions, but available evidence remains heterogeneous with respect to image acquisition, annotation strategy, model architecture, validation approach and clinical endpoints. Early reviews and meta-analyses summarized promising results, while more recent work has emphasized the need for external validation, standardized datasets, and prospective clinical implementation studies [14,25,26,27,28,29].

Several image- and video-based studies have demonstrated the technical feasibility of AI-assisted laryngeal lesion assessment. Wellenstein et al. demonstrated an AI-based system for the detection of laryngeal carcinoma during endoscopy, showing the potential for real-time application [15]. He et al. developed a convolutional neural network for laryngeal squamous cell carcinoma diagnosis based on laryngoscopic images [16]. Xiong et al. and Xu et al. reported deep-learning-based approaches for computer-aided laryngeal cancer diagnosis, whereas Paderno et al. addressed instance segmentation of upper aerodigestive tract cancers and demonstrated the relevance of site-specific performance [17,30,31].

The multicenter validation study by Sampieri et al. is particularly relevant to the interpretation of the present findings [32]. That study developed and externally validated a CADx model for classifying laryngeal lesions into high-risk and low-risk categories using laryngoscopy images from multiple tertiary referral centers. The strength of such a design is external validation and assessment of generalizability across institutions. By contrast, the present study evaluated a CE-MDR-certified AI system prospectively during real-time flexible laryngoscopy, albeit in a smaller single-center cohort. These approaches are complementary: retrospective multicenter image-based validation can assess model generalizability, whereas prospective real-time studies can assess feasibility under clinical workflow conditions.

### 4.5. Discordant Cases, Hyperkeratosis, and Non-Definitive AI Outputs

The discordant and non-definitive Zeno AI outputs provide important information about the current behavior of the system. In the present cohort, hyperkeratotic lesions were repeatedly upgraded by Zeno AI to malignant/suspicious. This finding should be interpreted in the context of the known diagnostic difficulty of preneoplastic and early malignant laryngeal lesions. The differentiation between hyperkeratosis, leukoplakia, low-grade dysplasia, high-grade dysplasia, carcinoma in situ, and early invasive carcinoma remains challenging even for experienced clinicians. Mannelli et al. emphasized that laryngeal preneoplastic lesions and early cancer represent a diagnostically difficult continuum [33].

From a safety perspective, false-positive upgrading may be less critical than missed or falsely reassuring classifications, because it may increase the likelihood of further diagnostic clarification. However, systematic upgrading can also reduce specificity and may lead to unnecessary concern, closer follow-up, a greater burden on the healthcare system or surgical intervention if AI output is overinterpreted. This is particularly relevant for vocal fold leukoplakia and hyperkeratotic lesions, where white-light morphology and NBI vascular patterns both contribute to risk stratification. Pietruszewska et al. showed that both plaque morphology and peripheral microvascular patterns are relevant for predicting high-risk vocal fold leukoplakia [34]. Therefore, suspicious AI output should be integrated with expert endoscopic assessment, NBI findings, clinical history and, where indicated, histopathology. If such lesions are going to be upgraded too frequently, this would reduce the benefits of potential AI use.

The uncertain category is equally important. In the present cohort, one histologically confirmed high-grade dysplasia was classified as uncertain by Zeno AI. This demonstrates that uncertain output should be interpreted as non-diagnostic rather than benign or reassuring. In clinical practice, uncertain AI output should prompt expert review, repeat high-quality visualization, NBI assessment, or histopathological clarification depending on the endoscopic appearance. Future AI systems should ideally distinguish between uncertainty caused by poor image quality, insufficient lesion representation, and true model uncertainty due to ambiguous lesion morphology.

### 4.6. Clinical Implications

The present results support the potential role of real-time AI analysis as an adjunct to flexible laryngoscopy, particularly as a second-reader or triage tool. In settings where laryngological expertise varies, such systems could in the future support the identification of patients who may require specialist evaluation, NBI assessment, microlaryngoscopy, or close follow-up. However, these potential applications were not evaluated in the present shadow-mode study and require prospective assessment with visible AI output and analyses stratified by examiner experience.

The current version of Zeno AI should not be regarded as an optical biopsy tool. Histopathology remains essential for definitive diagnosis, grading of dysplasia, confirmation of invasive carcinoma, and treatment planning [19]. The lack of differentiated lesion-specific classification also limits immediate clinical utility, because the distinction between papilloma, polyp, cyst, Reinke’s edema, granuloma, leukoplakia, dysplasia, carcinoma in situ, and invasive carcinoma is clinically relevant for counseling, urgency assessment, follow-up, and surgical planning.

Future versions of AI systems for laryngoscopy should therefore move beyond benign–malignant classification and incorporate lesion-specific classification, uncertainty calibration, and multimodal information from white-light endoscopy and NBI.

### 4.7. Limitations

This study has several limitations. First, it was conducted at a single tertiary center and included a limited histopathology subgroup. Accordingly, the diagnostic performance estimates have limited precision, as reflected by the wide confidence intervals. Diagnostic performance metrics against histopathology were therefore calculated only in patients with a clinical indication for microlaryngoscopy, resulting in selection bias and a higher pretest probability of relevant pathology. Nevertheless, it should be noted that this was a pilot study. Second, histopathology was not available for morphologically unremarkable cases or for most non-operated benign-appearing lesions. Consequently, agreement analyses in the full cohort used the expert majority assessment as a clinical reference standard rather than a histopathological gold standard. It should be emphasized that both the expert-based assessment and the clinical decision regarding which patients underwent microlaryngoscopy may have introduced bias into the study population and affected the diagnostic performance estimates. To reduce the influence of individual observer judgment, three experienced examiners independently classified each case, and the majority vote served as the expert reference. Nevertheless, expert consensus cannot replace histopathological verification.

Third, the AI output was not displayed to the examining physician. Therefore, the study did not evaluate whether AI assistance improves the performance of less experienced examiners, changes diagnostic confidence, or affects clinical management. Consequently, potential applications such as the use of AI as a triage or second-reader tool remain speculative and were not evaluated in the present study.

Fourth, cases classified as uncertain were excluded from the primary AI performance analysis. Although a sensitivity analysis was performed in which uncertain outputs were treated as test-negative for malignancy detection, exclusion of uncertain outputs may overestimate diagnostic performance.

Fifth, the system was evaluated within a structured acquisition setting, and post-epiglottic visualization was available in all lesion-positive cases and all cases with histopathology. Performance may differ under less standardized routine conditions or with lower-quality input data. The requirement for a stable three second output from the AI, in addition to the high-quality videos, may have resulted in more favorable performance estimates by preferentially retaining stable and readily classifiable findings. These criteria were applied to reduce the influence of transient artifacts caused by camera movement, mucus, loss of focus, or overexposure.

These factors may have influenced the observed agreement and diagnostic performance estimates. The structured acquisition protocol, exclusion of insufficient-quality videos, and previous glottic surgery may have produced more favorable results and may limit generalizability. Associated scarring may also have affected both expert interpretation and AI performance.

Finally, the study evaluated one AI system version only; further software development may alter classification behavior and generalizability.

These limitations underline that the present findings should be interpreted as evidence of feasibility and analytical performance under structured real-time conditions, rather than as proof of clinical benefit or replacement of histopathological diagnosis.

## 5. Conclusions and Outlook

In this prospective pilot study, Zeno AI showed high agreement with experienced examiners for real-time benign–malignant classification of glottic findings during structured flexible laryngoscopy. In the histopathology subgroup, diagnostic performance estimates were in the range of the expert majority assessment when only definitive AI outputs were considered. Importantly, no histopathologically confirmed malignant finding was classified as benign by Zeno AI. However, one histologically confirmed high-grade dysplasia was classified as uncertain, emphasizing that non-definitive AI outputs must be interpreted as non-diagnostic rather than reassuring and should prompt expert review without delay.

These findings support the feasibility of real-time AI analysis as a potential supporting tool in flexible laryngoscopy. A possible future application is its use as a triage or second-reader system, particularly in settings where examiner experience varies and where rapid prioritization of further diagnostic workup, specialist referral, or close follow-up is clinically relevant. However, because the AI output was not displayed to the examiner during the procedure, the present study evaluated analytical performance in a shadow-mode setting and did not assess the direct impact of AI assistance on clinical decision-making.

The current role of Zeno AI should therefore be understood as supportive risk stratification rather than autonomous diagnosis or optical biopsy. Histopathology remains essential for definitive diagnosis, grading of dysplasia, confirmation of invasive carcinoma, assessment of resection margins, adjuvant therapy planning, and molecular-pathological evaluation.

In terms of future development, AI-supported laryngoscopy may develop in several steps. A first generation of systems may support real-time benign–malignant risk stratification and triage during flexible laryngoscopy. A next developmental step could include differentiated lesion-specific classification, for example distinguishing papilloma, polyp, cyst, leukoplakia, dysplasia, carcinoma in situ, and invasive carcinoma. Such functionality could improve counseling, follow-up planning, surgical decision-making, and prioritization of microlaryngoscopy.

Beyond lesion-specific classification, future systems may integrate multimodal endoscopic information. Combining white-light endoscopy, narrow-band imaging, and functional information such as stroboscopy could support more individualized assessment of glottic lesions and improve preoperative risk stratification. In the longer term, integration of additional imaging modalities such as contact endoscopy or optical coherence tomography may further refine tissue characterization and intraoperative orientation. Such developments could bring AI-supported endoscopy closer to the concept of optical biopsy, particularly for mapping suspicious mucosal areas, assessing resection margins, and grading precancerous changes. However, these applications remain future perspectives and require dedicated clinical validation.

Future clinical studies should evaluate visible AI assistance under routine clinical conditions and assess its impact on diagnostic confidence, examiner performance, referral decisions, treatment planning, and patient-relevant outcomes. Larger multicenter cohorts with increased histopathological reference, external validation, and follow-up of morphologically unremarkable and non-operated findings will be required to define the clinical value and limitations of AI-supported flexible laryngoscopy.

## Figures and Tables

**Table 1:** Patient and cohort characteristics.

Characteristic	Full Cohort, n = 109	Lesion-Positive Cases, n = 45	Histopathology Subgroup, n = 36
Male sex	63/109 (57.8%)	30/45 (66.7%)	25/36 (69.4%)
Female sex	46/109 (42.2%)	15/45 (33.3%)	11/36 (30.6%)
Mean age, years	61.3	59.1	59.7
Age range, years	4–86	4–84	4–84
Previous glottic surgery	—	23/45 (51.1%)	19/36 (52.8%)

**Table 2:** Endoscopic acquisition characteristics.

Acquisition Characteristics	Full Cohort, n = 109	Lesion-Positive Cases, n = 45	Histopathology Subgroup, n = 36
Post-epiglottic visualization	106/109 (97.2%)	45/45 (100%)	36/36 (100%)
Near-contact imaging	60/109 (55.0%)	38/45 (84.4%)	31/36 (86.1%)
Rotated/inverted glottic visualization	52/109 (47.7%)	31/45 (68.9%)	26/36 (72.2%)
Evaluable NBI sequence	65/109 (59.6%)	41/45 (91.1%)	35/36 (97.2%)

**Table 3:** Distribution of expert majority and AI classifications in the full cohort.

Classification	Expert Majority, n (%)	Zeno AI, n (%)
Morphologically unremarkable	64/109 (58.7%)	65/109 (59.6%)
Definitive lesion-positive output	45/109 (41.3%)	38/109 (34.9%) *
Benign lesion	34/109 (31.2%)	26/109 (23.9%)
Malignant/suspicious lesion	11/109 (10.1%)	12/109 (11.0%)
Uncertain	—	6/109 (5.5%)

* For Zeno AI, definitive lesion-positive output includes benign and malignant outputs; uncertain outputs were reported separately.

**Table 4:** Cross-tabulation of expert majority and Zeno AI classification in cases with definitive AI output (n = 103).

Expert Majority\Zeno AI	AI Unremarkable	AI Benign	AI Malignant
Morphologically unremarkable	63	0	0
Benign	2	26	2
Malignant/suspicious	0	0	10

**Table 5:** Discordant and non-definitive AI outputs, n = 10.

Expert Majority	Expert Suspected Diagnosis	Zeno AI Output	Pattern
Benign	Hyperkeratosis	Malignant	Upgrading
Benign	Hyperkeratosis	Malignant	Upgrading
Benign	Vocal fold cyst	Unremarkable	Not detected
Benign	Papilloma	Unremarkable	Not detected
Benign	Papilloma	Uncertain	Non-definitive
Benign	Papilloma	Uncertain	Non-definitive
Benign	Vocal fold polyp	Uncertain	Non-definitive
Benign	Hyperplastic vessels	Uncertain	Non-definitive
Unremarkable	Morphologically unremarkable	Uncertain	Non-definitive
Malignant/suspicious	Carcinoma	Uncertain	Non-definitive

**Table 6:** Histopathological findings in the histopathology subgroup, n = 36.

Histological Diagnosis	n = 36	Histopathological Category
Papilloma	11	Negative for malignancy
Reinke’s edema	3	Negative for malignancy
Cyst	5	Negative for malignancy
Granuloma	1	Negative for malignancy
Polyp	2	Negative for malignancy
Low-grade dysplasia	2	Negative for malignancy
Hyperkeratosis	1	Negative for malignancy
Granulation tissue	1	Negative for malignancy
Sarcoidosis	1	Negative for malignancy
High-grade dysplasia	3	Positive for malignancy
Carcinoma in situ (CIS)	1	Positive for malignancy
Squamous cell carcinoma (SCC)	5	Positive for malignancy

**Table 7:** Diagnostic performance compared with histopathology.

Analysis	n	Sensitivity	Specificity	PPV	NPV	Cohen’s κ
Expert majority vs. histopathology	36	100%	96.3%	90.0%	100%	0.929
AI vs. histopathology, primary intent-to-diagnose analysis	36	88.9%	92.6%	80.0%	96.2%	0.786
AI vs. histopathology, secondary definitive-output analysis	32	100%	91.7%	80.0%	100%	0.846

## Data Availability

The datasets generated and analyzed during the current study are not publicly available due to patient privacy and data protection regulations but are available from the corresponding author upon reasonable request. Representative endoscopic video sequences are provided as Supplementary Materials.

## Supplementary Materials

The following supporting information can be downloaded at https://www.mdpi.com/article/10.3390/medsci14040458/s1. Supplementary Video S1: Flexible laryngoscopy of a glottic laryngeal papilloma with Zeno AI evaluation. Supplementary Video S2: Flexible laryngoscopy of two vocal fold polyps with Zeno AI evaluation. Supplementary Video S3: Flexible laryngoscopy of a high-grade dysplasia classified as uncertain by Zeno AI. File S4: Supplementary Information on the Development and Internal validation of Zeno AI.

## Abbreviations

The following abbreviations are used in this manuscript:

AI	Artificial intelligence
ELS	European Laryngological Society
PPV	Positive predictive value
NPV	Negative predictive value
NBI	Narrow-band imaging
CIS	Carcinoma in situ
