# Supplementary material for: Real-Time AI-Based Assessment of Glottic Lesions During Flexible Laryngoscopy: Comparison with Expert Evaluation and Histopathology
Source: Med Sci (Basel). 2026 Aug 6;14(4):458. doi: 10.3390/medsci14040458 (PMC13515216; doi:10.3390/medsci14040458)
Supplement: Supplementary file 1 [file medsci-14-00458-s001.zip › File S4.pdf]

Supplementary Information on the Development and Internal validation of Zeno AI

The CE-MDR Class IIb-certified Zeno AI system (version 1.4.0) was developed through a collaboration between three hospitals—University Medical Center Groningen (UMC Groningen), Radboud University Medical Center (Radboud UMC), and Martini Ziekenhuis Groningen—and WSK Medical, a company specializing in deep-learning applications for medical use.

The development and internal validation of the system are described in detail by Krijnen et al.:
Krijnen, H. K., van Rhee, N. F., Wilmes, C. M. L. H., Hofman, M., Woodburn, J., San Giorgi, M. R. M., Schoonbeek, R. C., Wegner, I., Wellenstein, D. J., van den Broek, G. B., Halmos, G. B., & Plaat, B. E. C. (2026). Development of an artificial intelligence model to improve laryngeal lesion diagnostics. Manuscript submitted for publication.
